# Two Hits of EDCs Three Generations Apart: Effects on Social Behaviors in Rats, and Analysis by Machine Learning

**DOI:** 10.3390/toxics10010030

**Published:** 2022-01-11

**Authors:** Ross Gillette, Michelle Dias, Michael P. Reilly, Lindsay M. Thompson, Norma J. Castillo, Erin L. Vasquez, David Crews, Andrea C. Gore

**Affiliations:** 1 Division of Pharmacology and Toxicology, College of Pharmacy, The University of Texas at Austin, Austin, TX 78712, USA; rossg@austin.utexas.edu (R.G.); michelledias10@gmail.com (M.D.); michaelreillyp@gmail.com (M.P.R.); lindsay.thompson82@utexas.edu (L.M.T.); norma.j.castillo95@utexas.edu (N.J.C.); erinvasquez6@gmail.com (E.L.V.); 2 Department of Integrative Biology, The University of Texas at Austin, Austin, TX 78712, USA; crews@mail.utexas.edu

**Keywords:** endocrine-disrupting chemicals (EDC), Aroclor 1221 (A1221), PCBs, vinclozolin, social behavior, sex differences, transgenerational, epigenetic

## Abstract

All individuals are directly exposed to extant environmental endocrine-disrupting chemicals (EDCs), and indirectly exposed through transgenerational inheritance from our ancestors. Although direct and ancestral exposures can each lead to deficits in behaviors, their interactions are not known. Here we focused on social behaviors based on evidence of their vulnerability to direct or ancestral exposures, together with their importance in reproduction and survival of a species. Using a novel “two hits, three generations apart” experimental rat model, we investigated interactions of two classes of EDCs across six generations. PCBs (a weakly estrogenic mixture Aroclor 1221, 1 mg/kg), Vinclozolin (antiandrogenic, 1 mg/kg) or vehicle (6% DMSO in sesame oil) were administered to pregnant rat dams (F0) to directly expose the F1 generation, with subsequent breeding through paternal or maternal lines. A second EDC hit was given to F3 dams, thereby exposing the F4 generation, with breeding through the F6 generation. Approximately 1200 male and female rats from F1, F3, F4 and F6 generations were run through tests of sociability and social novelty as indices of social preference. We leveraged machine learning using DeepLabCut to analyze nuanced social behaviors such as nose touching with accuracy similar to a human scorer. Surprisingly, social behaviors were affected in ancestrally exposed but not directly exposed individuals, particularly females from a paternally exposed breeding lineage. Effects varied by EDC: Vinclozolin affected aspects of behavior in the F3 generation while PCBs affected both the F3 and F6 generations. Taken together, our data suggest that specific aspects of behavior are particularly vulnerable to heritable ancestral exposure of EDC contamination, that there are sex differences, and that lineage is a key factor in transgenerational outcomes.

## 1. Introduction

We live in a world that is irreversibly contaminated as a consequence of the chemical revolution that began in the 1940s. The industrial, agricultural, and pharmaceutical industries, to name only a few, have produced hundreds of thousands of chemicals, among which nearly 1000 are now classified as endocrine-disrupting chemicals (EDCs) [1,2]. The consequences of EDC exposure are manifested as endocrine and neurological disorders in individuals directly exposed, especially during sensitive life stages such as fetal development. Furthermore, exposure can cause disease and dysfunction for multiple generations without additional exposure due to heritable epigenetic mechanisms [3,4]. Thus, the complex diseases and dysfunctions associated with EDCs represent the interaction of historical and contemporary exposures. The complexities arising from nearly a century of EDC exposure—about five generations in humans and hundreds of generations in rodents —must be studied in a laboratory setting if there is any hope that we can anticipate similar issues arising in humans.

Among those phenotypes affected by EDCs are social behaviors. These behaviors allow individuals to identify and distinguish others in or outside of their species, serve to establish cohesive social structure and hierarchies, provide cues necessary for parental and sexual behaviors, and are critical for the survival of a species. Several EDC classes including polychlorinated biphenyls (PCBs), vinclozolin, bisphenol A (BPA), phthalates, and chlorpyrifos cause changes in social behaviors in rodents [5,6,7,8,9,10,11,12]. In fact, behaviors, particularly those that are hormone-sensitive such as social behavior, appear to be among the most sensitive to EDCs [13,14]. Beyond these studies on direct exposure are those, although fewer in number, that have demonstrated intergenerational EDC effects on social behaviors [15,16,17,18,19]. However, there has been no work, to our knowledge, on the interactions of ancestral and direct exposures, a gap in understanding of the current real-world dilemma.

Neurobehavioral research must be conducted in the context of sex differences and parental lineage of origin based on strong evidence for sexually dimorphic effects of EDCs, and the importance of maternal vs. paternal exposure on phenotypic outcomes [20]. However, the sheer numbers of animals necessitated by multigenerational breeding and testing of both sexes through parental lineages, and the labor necessary to score nuanced social behaviors such as nose-to-nose interactions of rodent conspecifics, has made large-scale social behavioral experiments prohibitive. Yet, it is these difficult-to-observe behaviors that provide the most salient information about a potential mate that are the most ethologically important [21]. Advances in computer vision and object classification and recognition have established the tools necessary for using machine learning to automate the identification of complex animal behavior [22,23]. These tools were developed and applied to the current analysis.

Here, we used two different EDC classes selected for differences in their historical usage and in their mechanism of action. Polychlorinated biphenyls (PCBs) represent a legacy group of EDCs that were widely used until their ban in the United States and elsewhere in the 1970s. The industrial PCB mixture used herein, Aroclor 1221 (A1221), acts mainly through estrogenic signaling pathways [24]. Vinclozolin (VIN) is in modern use as a fungicide and is primarily antiandrogenic in its action [25,26]. Both VIN [27] and PCBs [28] at high dosages induce overt reproductive toxicity, and at lower concentrations, especially during development, act as EDCs to perturb hormones and their actions [14]. Each has been characterized for its neurobehavioral consequences both for direct and ancestral exposure [1,6,14,19,29,30,31,32] but not for their interactions across generations. This is a particularly important but untested concept considering the real-world scenario that humans and animals today were likely exposed to high levels of PCBs 50 years ago, leading to a potentially heritable “imprint,” and now their descendants are subjected to exposures to modern classes of EDCs such as VIN.

## 2. Materials and Methods

### 2.1. Animals and Treatment

All animal work was conducted using humane procedures that were approved by the Institutional Animal Care and Use Committee at The University of Texas at Austin in accordance with NIH guidelines. Three month old male and female Sprague-Dawley rats were purchased from Envigo (Envigo, Indianapolis, IN, USA) and acclimated to the animal housing facility and light cycle (14:10 dark:light) for two weeks. All rooms were kept at a consistent temperature (22 C) and all rats had ad libitum access to filtered tap water and a low phytoestrogen diet. (Teklad 2019: Envigo, Indianapolis, IN, USA).

**Figure 1:**
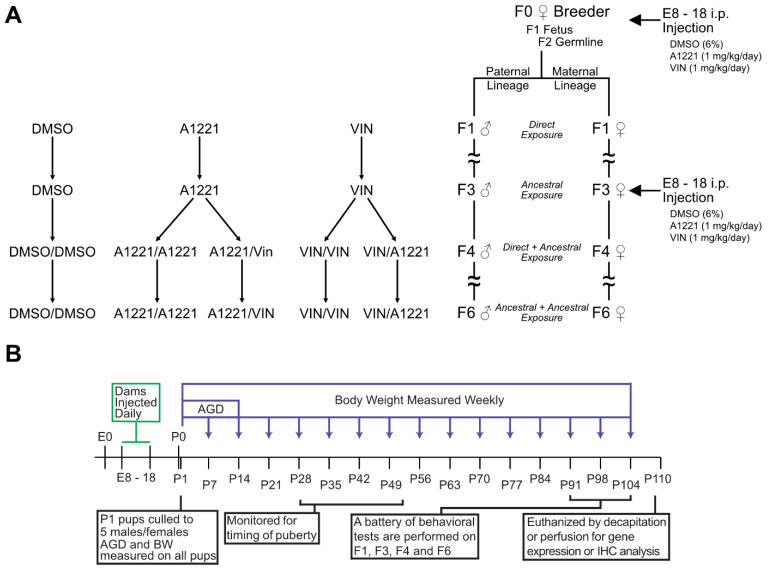
Experimental design and timeline. The experimental design is shown (**A**) including parental lineages, generations, and treatment groups used for analysis. EDC exposure was administered via i.p. injection to F0 and F3 pregnant dams from E8-18. The timeline (**B**) indicates all experimental manipulations and measurements taken. AGD: anogenital distance.

The vaginal cytology of virgin breeder females was observed daily the week prior to mating. On the day of proestrus, females (F0) were paired overnight with a sexually experienced male rat and observed under red light for copulatory behaviors. If the female displayed receptive behaviors, the pair was left overnight with food and water. The next morning, the presence of sperm in a vaginal smear was used to confirm pregnancy and marked as embryonic day 1 (E1). Pregnant F0 dams were randomly assigned to one of three treatment groups (Table 1): vehicle (6% DMSO in sesame oil), Aroclor 1221 (A1221, 1 mg/kg), or Vinclozolin (VIN, 1 mg/kg). The dosages and route were selected to match previous work and to fall within ranges of human exposures [33,34,35,36]. The investigators were blind to treatments throughout the study, with the code broken only when all experimental work was completed. Pregnant dams were weighed and injected daily via i.p. injection from E8–E18 two hours prior to lights out. This age range was selected to encompass a prenatal period when germline epigenetic marks are established, as well as the beginning of the critical period of brain sexual differentiation [37,38]. The i.p. route also matched prior work, although current studies in the lab have switched to feeding EDCs, with similar results, to better approximate the route of most human exposures. At E18, dams were given nesting material and left undisturbed until birth (referred to as P0). On the day after birth, postnatal day 1 (P1), each litter was culled to 5 males and 5 females per litter based on median anogenital index (anogenital distance divided by the cube root of bodyweight) to maintain an equivalent sex ratio between all litters. At P21, pups were weaned into separate cages with 2–3 same-sex littermates per cage. Of the five males and females retained in each litter, 2–3 of each sex were used for behavioral testing. On P80, one male and one female from each litter that had not been used for behavioral testing was mated with an untreated animal to generate the F2 paternal and maternal lineages, respectively. The breeding protocol is shown in Figure 1.

In the resulting F2 generation, one P80 female from each of the maternal litters was bred with an untreated male to continue the maternal lineage, and one P80 male from each paternal litter was bred with an untreated female to continue the paternal lineage. All untreated male and female breeders were purchased from Envigo, delivered to the lab at P60, and were allowed 2 weeks of habituation before breeding began. This same maternal and paternal breeding paradigm was used until the sixth (F6) generation.

To ascertain interactions of direct and transgenerational EDC exposures, a subset of F3 females from the maternal lineage was bred with untreated males, and a group of naïve females purchased from Envigo was bred with a subset of F3 males from the paternal lineage (Figure 1A). Exposure to EDCs was performed identically as in the F1 generation. Because of the large number of possible combinations, the two hits across three generations (F1/F4) were limited to VEH/VEH, A1221/A1221, A1221/VIN, VIN/VIN, and VIN/A1221 (Figure 1A).

A total of 306 litters were used across all treatment groups with an average N~10 litters per generation/lineage/treatment. The total number of litters used for each generation, lineage, sex, and treatment are shown in Table 1, with approximately 20 individuals per generation/lineage/sex/treatment and a total of 1209 behaviorally characterized animals (Table 1). The litters and resulting behaviorally characterized individuals were spread across 7 cohorts that spanned 4 years.

### 2.2. Sociability Apparatus and Behavioral Analysis

Each individual animal was run through a battery of behavioral tests beginning on P90: Ultrasonic Vocalizations (USV), and Mate Preference (MP), Open Field (OF), Sociability, Social Novelty, Light/Dark box (LD), Elevated Plus (EP). The order of behavioral tasks was the same for all animals (as listed above) and each behavioral task was separated by 48 h and always occurred between 1 and 4 h after lights off under dim red light. All animals were transported to the behavioral analysis rooms in black-out covered carts as to prevent light pulse exposure. In the current manuscript, data from the sociability and social novelty tasks are presented. One week after completing the behavior tests, animals were euthanized by rapid decapitation (Figure 1B).

**Figure 2:**
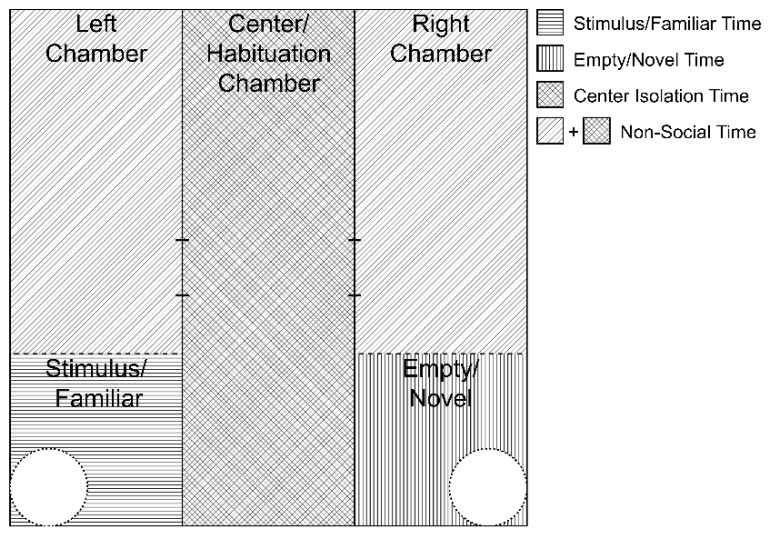
Sociability apparatus. A diagram of the three-chambered sociability apparatus is shown where the fill pattern indicates the various sections of the apparatus that were digitally segmented for analysis. The test occurred in 3 distinct phases. The first (habituation—5 min) occurred with the experimental rat placed in the center chamber (cross-hatched) with the doors to the left and right chambers shut such that the experimental animal could not access either side arena. During the subsequent two stages (sociability—10 min and social novelty—10 min) the doors were opened, allowing the experimental rat free access to the three chambers. The far corners of the side chambers each held a cylindrical holding chamber, with or without a rat contained within. For scoring purposes, the center chamber was scored as social isolation time. Total nonsocial time was calculated by adding the time spent in the distal left and right chambers (diagonal) with the time spent in the center chamber. Time spent near either the stimulus/familiar animal (horizontal) or empty cage/novel animal (vertical) was scored only when the target experimental animal’s center of mass was in close proximity (less than approximately one body length) to the respective enclosure.

A three-chambered sociability chamber (Stoelting, AnyMaze) [39] was used as published and according to the methodology previously published by our lab [6,19,31]. The chamber consists of a 100 cm (wide) × 100 cm (long) Plexiglas square partitioned into 3 equivalent chambers approximately 33 cm (wide) by 100 cm (long), with a door measuring 10 cm × 11 cm leading to the middle chamber (Figure 2). The left and right chambers included a cylindrical stimulus animal enclosure in the bottom corners of the arena that was 15 cm wide and had vertical metal rods separated by 1 cm that allowed facial investigation and nose touching but prevented more extensive interactions.

A sociability trial consisted of 3 distinct stages: habituation (5 min), sociability (10 min), and social novelty (10 min). In the habituation phase, an animal was placed in the center chamber with the entrances blocked such that the target experimental animal could not access the left or right chambers. A same sex- and age-matched stimulus animal was placed in one stimulus enclosure and randomly positioned in either the left- or right-flanking chamber. An empty stimulus enclosure was placed in the opposite flanking chamber. Doors were opened at the beginning of the sociability phase, during which the experimental animal was allowed to freely explore the arena and investigate the stimulus animal or empty stimulus enclosure. At the end of the sociability phase the target experimental animal was temporarily removed from the apparatus and a novel stimulus animal was placed in the empty stimulus enclosure. The placement of the enclosures containing the familiar and the novel stimulus rats on the left or right was random to avoid direction or side biases. During the social novelty phase, the target experimental animal was returned to the center portion of the arena and again allowed to freely explore the arena for 10 min and investigate the familiar and the novel stimulus animal. At the end of the social novelty phase, each animal was returned to their respective home cage. Stimulus rats were used in no more than 3 trials in one day to avoid behavioral changes due to repeated testing. All female experimental and stimulus animals were used for testing while in diestrus.

All behavioral trials were analyzed in real time with AnyMaze software (Stoelting), as published [6,19,31]. The testing arena was digitally segmented into left, center, and right chambers. An additional digital segment was drawn around the stimulus enclosure on each side (approximately 33 cm × 33 cm—Figure 2) to indicate proximity of the target experimental animal to the stimulus enclosures. An animal was considered to enter an area if 80% of the animal’s area was inside of that area, which is equivalent to an animal having 4 paws within the area.

### 2.3. Data Exclusion Criteria

Occupancy plots showing animal position across the duration of a trial were extracted from AnyMaze and visually analyzed for errors in tracking; the latter were marked for retracking in an attempt to rescue the data. After retracking, the occupancy plots were analyzed again. Instances that did not show improved tracking were excluded from analysis. Further trials were removed from analysis for various technical reasons (incomplete habituation period, animals that escaped the arena, and animals that altered the position of stimulus restraint chambers). Raw data were then extracted from AnyMaze and checked for obvious anomalies in the distance traveled to indicate poor tracking fidelity. Trials that had lost more than 20 s of experimental time due to video retracking were also excluded from analysis. Finally, stage 1 of sociability (Stimulus vs. Empty Chamber) was analyzed to determine trials in which the experimental animal did not visit each flanking chamber for at least 10 s. These trials were left as part of the analysis for stage 1 but were removed from stage 2 (Familiar vs. Novel) of sociability because these experimental rats would not have had the opportunity to become familiar with the stimulus animal, making a choice between a “familiar” and a novel rat moot.

### 2.4. Nose Touch Detection with Machine Learning and DeepLabCut

**Figure 3:**
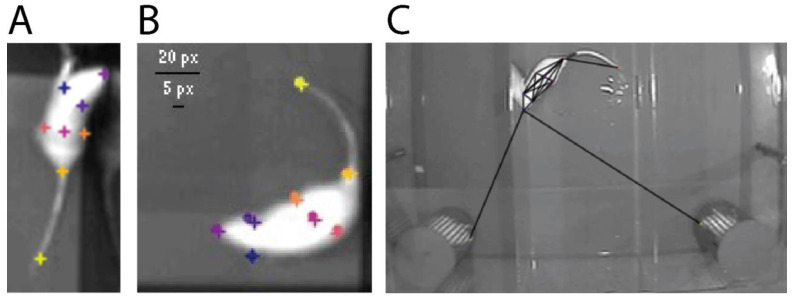
Automated animal tracking for nose-touch detection. Representative images of the training process produced in DeepLabCut are shown. (**A**) The 8 user-labeled body parts (nose, left and right ears, left and right flank, body center, tail base, and tail tip) are indicated on an image (+) used to train the model to identify each individual body part; (**B**) a test image showing the difference in placement between user-labeled body parts (**+**) and the location of the same body part (•) predicted by the model; (**C**) the final pose-estimation model demonstrating the automated body-part tracking in an experimental video where the noses of two stimulus animals are simultaneously tracked in relation to the body and nose of the experimental animal.

Facial investigation and nose touches are a critical aspect of rodent social interaction and investigation [6,32,40] but they are also the most labor-intensive part of the analysis, as they involve an investigator iteratively viewing and scoring every recorded trial of the sociability and the social novelty tests from thousands of trials. To automate the detection of nose touches from recorded behavioral trials we used DeepLabCut (version 2.2b [41]) which employs deep residual neural networks to predict the location of individual body parts. A total of 16 trial videos and 20 frames from each video (320 total frames) were used to create a training dataset in which an experimenter manually labeled 8 individual body parts (nose, left and right ears, left and right flank, body center, tail base, and tail end) of the target experimental animal and the nose and tail of the stimulus animals (Figure 3). The manually labeled frames were split into a training set (95%) and test set (5%) and the network was trained for 250K iterations. The resulting body part position data were then used to calculate the distance between the nose of the target experimental animal and the two stimulus animals for every frame of each video. A nose touch was then marked when the distance between the experimental and stimulus animals’ noses was below a specified threshold. Nose touch instances and duration were then calculated by employing run-length encoding, in which consecutive video frames in which a nose touch was detected were grouped together as a nose touch instance. A second and separate human-scored validation dataset was then used to optimize the distance between two noses and the time within that distance that most closely represented human scoring. We determined that a distance of 7.5 pixels between the experimental and stimulus animal’s noses and 200 consecutive milliseconds (about 7 video frames) within that distance most closely matched what a human scorer considered a nose touch. Finally, video frames in which the machine learning model displayed low certainty (*p* < 0.90) of the position of either the experimental animal’s or stimulus animal’s nose were excluded from analysis.

Once validated, the trained model was applied to all behavioral videos, nose-touch instances were extracted using the parameters determined above, and the results were analyzed identically to the statistical methods used for the other behavioral metrics described above. A third and separate validation video set (16 videos) was randomly selected from all of the videos processed with the final model, again hand-scored by a blind experimenter, and compared to the machine-learning model to determine the accuracy of the model against a dataset that was entirely removed and separate from the optimization process. This third validation set was used to determine if the model generalized well or was overfit to the datasets used to train the machine learning model or optimize the detection of nose touches.

### 2.5. Statistics

All statistical analyses were performed in *R* (version 4.0.2—[42]) with the base *stats* package (version 4.0.2). Figures were created with *ggplot2* (version 3.3.3), and were edited only for style with Adobe Illustrator (CS5). Behavioral metrics were checked for normality (Shapiro–Wilk Test [43]) and homogeneity of variance (Bartlett’s Test [44]) using R’s base *stats* package. As is typical of large behavioral datasets, most of the individual metrics violated these assumptions that are required for the traditional application of parametric statistics. To determine sex differences the raw data were analyzed with a Kruskal–Wallis one-way analysis of variance [45] within generation and parental lineage where all treatments were collapsed into sex. Effect size was calculated for sex differences using Cohen’s D [46] and reported with each statistic. To determine effects of EDC treatment, individually optimized Box–Cox power transformations (R—*EnvStats* version 2.4.0 [47]) were applied to all behavioral metrics separately but equally across all generations, lineages, sexes, and treatments within a metric. As large differences were expected due to sex differences in behavioral tests, one-way ANOVAs (R—*car* version 3.0-10 [48]) were applied within sex to determine effects of treatment (e.g., EDC exposure) and considered significant if *p* < 0.05. The effect size, or the amount of variance accounted for by the linear model, was calculated as partial-eta-squared (R—*lsr* version 0.5 [49]) for each significant effect and reported with the corresponding statistic. A partial-eta-squared value of 0.01 is considered small, 0.09 is considered medium, and 0.25 is considered large. If an ANOVA were determined significant, Tukey’s Honest Significant Difference [50] pairwise post hoc tests were applied to determine individual group difference and *p*-values were appropriately adjusted for multiple comparisons.

## 3. Results

### 3.1. Sociability

#### 3.1.1. Sex Differences

Given the large sample size due to our experimental design, we were afforded the unique opportunity to characterize sex differences inherent to the Sociability test with data collapsed across all other variables (treatment, generation, lineage). Previous studies show that sex differences are large with a bimodal distribution, leading us to use nonparametric tests (Kruskal–Wallis H-test) on an N of ~600 per group. The social behavior data are summarized in Table 2.

Females were more active and traveled farther in the 10 min trial than did males (*H*(1) = 556.17, *p* < 0.0001, *d* = 1.72). Females showed a reduced social preference score compared to males, (*H*(1) = 45.15, *p* < 0.0001, *d* = 0.31) and spent less time near the stimulus animal (*H*(1) = 29.39, *p* < 0.0001, *d* = 0.31) and more time near an empty stimulus enclosure (*H*(1) = 28.52, *p* < 0.0001, *d* = 0.20). Females visited both the stimulus animal (*H*(1) = 60.7, *p* < 0.0001, *d* = 0.47) and the empty enclosure more often than males (*H*(1) = 198.53, *p* < 0.0001, *d* = 0.88). However, the females’ average visit time to both the stimulus animal (*H*(1) = 83.79, *p* < 0.0001, *d* = 0.50) and the empty enclosure were shorter than those in males (*H*(1) = 24.69, *p* < 0.0001, *d* = 0.38). Overall, females spent slightly more time in nonsocial areas of the apparatus (*H*(1) = 7.11, *p* = 0.0077, *d* = 0.15), but males spent more time in the isolated center chamber than did females (*H*(1) = 11.15, *p* < 0.0008, *d* = 0.24).

#### 3.1.2. Effect of EDC Exposure

**Figure 4:**
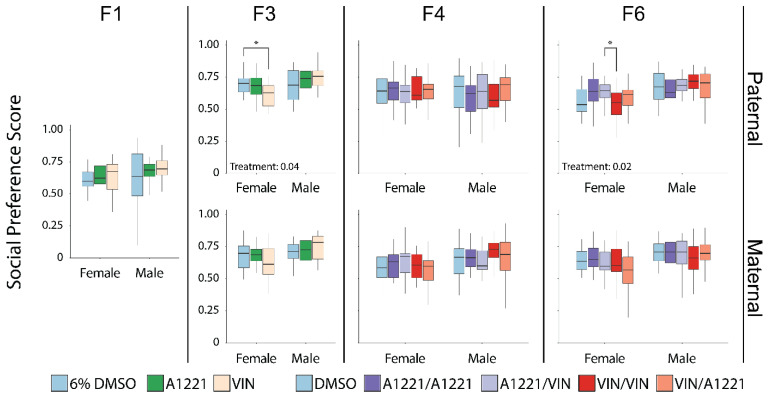
In the sociability test, the social preference score (time spent investigating the stimulus animal divided by the sum of the time spent investigating both the stimulus animal and an empty enclosure) for sociability is graphed as box and whisker plots (minimum, 25% quartile, median, 75% quartile, and maximum) shown separately by generation (F1 to F6 from left to right), lineage (paternal—top and maternal—bottom), and sex (indicated on *x*-axis). Lineage does not apply to the F1 generation. The social preference score was calculated as the time spent near the stimulus animal divided by the time spent near the stimulus animal plus the time spent near the empty enclosure. Scores above 0.5 indicate a preference for socializing with the stimulus animal. All group means were above the 0.5 threshold. Significant post hoc tests as determined by Tukey HSD are indicated with bars connecting the respective groups (* <0.05).

Prenatal EDC exposure did not affect locomotion (total distance traveled) in any generation, breeding lineage, or sex. We calculated a social preference score as the time spent investigating the stimulus animal divided by the sum of the time spent investigating both the stimulus animal and an empty enclosure to allow a direct comparison of preference that encompassed the two choices presented to the target animal. Direct exposure to prenatal EDCs (F1 generation) did not affect social preference or other more nuanced behavioral metrics (time spent with the stimulus animal, visits, or nonsocial time). In the F3 generation, which had only ancestral and not direct EDC exposure, there was a significant effect of treatment on the social preference score in females of the paternal lineage (*F*(2,60) = 3.54, *p* = 0.04, η*p*^2^ = 0.11—Figure 4), driven by a decrease in social preference due to ancestral VIN exposure (*p* = 0.04). Other endpoints were unaffected in the paternal lineage. In the F3 maternal lineage, the only metric affected was the average time females spent visiting an empty stimulus chamber (*F*(2,60) = 3.54, *p* = 0.04, η*p*^2^ = 0.11—data not shown).

**Figure 5:**
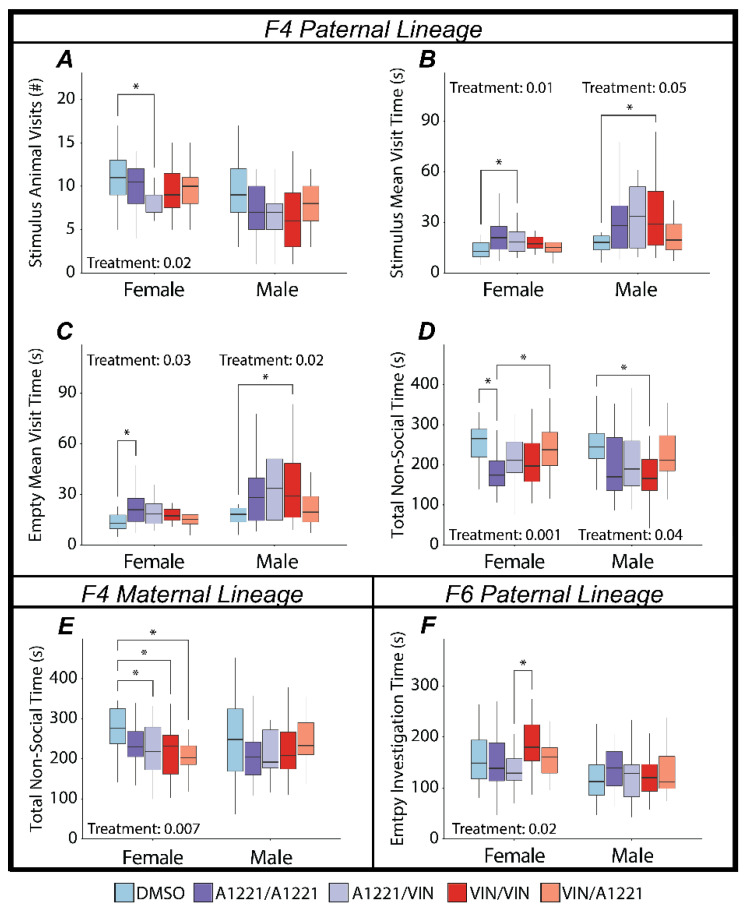
Aspects of social interaction dynamics in the test for sociability are graphed as box and whisker plots (minimum, 25% quartile, median, 75% quartile, and maximum). Main effects of treatment were determined by a one-way ANOVA within each sex and lineage, and indicated for *p* < 0.05. Significant post hoc results as determined by Tukey HSD and appropriately adjusted for multiple comparisons are indicated with bars connecting the respective groups (* <0.05). Shown are significant results for the F4 Paternal lineage (**A**–**D**), the F4 maternal lineage (**E**), and the F6 Paternal Lineage (**F**).

In the F4 generation, which represents ancestral exposure (first hit) combined with a second hit of direct fetal exposure three generations later, social preference was not affected by treatment in either sex or lineage. In females, other behaviors were affected, including the number of visits to the arena containing a stimulus animal (*F*(4,95) = 2.94, *p* = 0.02, η*p*^2^ = 0.11—Figure 5A). This effect was driven primarily by a decreased number of visits by the A1221/VIN females (*p* = 0.009) compared to DMSO vehicle. This seems to have been compensated for by an increased mean visit time to the stimulus animal (*F*(4,95) = 3.91, *p* = 0.01, η*p*^2^ = 0.14—Figure 5B), again driven by the A1221/VIN group vs. DMSO (*p* = 0.01). An effect on the mean visit time to the empty enclosure was also identified (*F*(4,91) = 2.80, *p* = 0.03, η*p*^2^ = 0.1—Figure 5C), in this case driven by an increase in visits by the A1221/A1221 females compared to DMSO vehicle (*p* < 0.01). The same A1221/A1221 females showed a decrease in total nonsocial time (*F*(4,91) = 4.81, *p* = 0.001, η*p*^2^ = 0.17—Figure 5D) compared to DMSO (*p* < 0.01) and VIN/A1221 (*p* = 0.02).

Males from the F4 paternal lineage showed few effects with the sole exception of the VIN/VIN treatment group compared to vehicle, as seen for mean visit time to the stimulus animal (*F*(4,91) = 2.49, *p* = 0.05, η*p*^2^ = 0.10—Figure 5B) and the empty enclosure (*F*(4,91) = 3.00, *p* = 0.02, η*p*^2^ = 0.12—Figure 5C) in which the VIN/VIN males showed increase duration visits to both (*p* = 0.05 and *p* = 0.03), respectively compared to DMSO vehicle. This increase in mean visit time was accompanied by a decrease in total nonsocial time (*F*(4,91) = 2.66, *p* = 0.04, η*p*^2^ = 0.10—Figure 5D) by VIN/VIN compared to vehicle males (*p* = 0.02).

A single effect of treatment was identified in the maternal lineage females of the F4 generation, for which total nonsocial time (*F*(4,91) = 3.76, *p* = 0.007, η*p*^2^ = 0.14—Figure 5E) was reduced in the A1221/VIN (*p* = 0.04), VIN/A1221 (*p* < 0.01), and VIN/VIN (*p* = 0.03) groups compared to DMSO.

Treatment effects on the social preference in the F6 generation, which represents two cumulative ancestral exposures three generations apart, were exclusive to females from the paternal lineage (*F*(4,95) = 3.07, *p* = 0.02, η*p*^2^ = 0.11—Figure 4). The A1221/VIN group showed a marginally increased preference for social affiliation compared to controls (*p* = 0.08) and this score was significantly greater in A1221/VIN than the VIN/VIN group (*p* = 0.04). This effect was accompanied by an inverse relationship in time investigating the empty enclosure (A1221/VIN < VIN/VIN; (*F*(4,95) = 3.05, *p* = 0.02, η*p*^2^ = 0.11—Figure 5F).

### 3.2. Social Novelty

#### 3.2.1. Sex Differences

We again leveraged the large sample size from our experiments to establish a definitive sex differences profile characteristic of social novelty (Table 2). Females traveled a greater distance in the 10 min trial than males (*H*(1) = 533.72, *p* < 0.0001, *d* = 1.67). Females also showed a modest increase in social novelty score over males (*H*(1) = 4.28, *p* = 0.039, *d* = 0.14), spent less time with the familiar stimulus animal (*H*(1) = 18.04 *p* < 0.0001, *d* = 0.29), but there was no difference in the time spent with the novel stimulus animal (*H*(1) = 0.70, *p* = 0.403, *d* = 0.09). Females displayed more visits to both the familiar stimulus animal (*H*(1) = 122.81, *p* < 0.0001, *d* = 0.68) and the novel stimulus animal (*H*(1) = 164.05, *p* < 0.0001, *d* = 0.81) but spent less average time per visit with the familiar (*H*(1) = 144.24, *p* < 0.0001, *d* = 0.55) and novel stimulus animals than males (*H*(1) = 104.46, *p* < 0.0001, *d* = 0.61). Overall, females had less total social time, the time associating with either the novel or familiar stimulus animal (*H*(1) = 31.89, *p* < 0.0001, *d* = 0.33), more nonsocial time (*H*(1) = 24.03, *p* < 0.0001, *d* = 0.25), and more time isolated in the center chamber of the apparatus (*H*(1) = 19.07, *p* < 0.0001, *d* = 0.20).

#### 3.2.2. Effect of EDC Exposure

**Figure 6:**
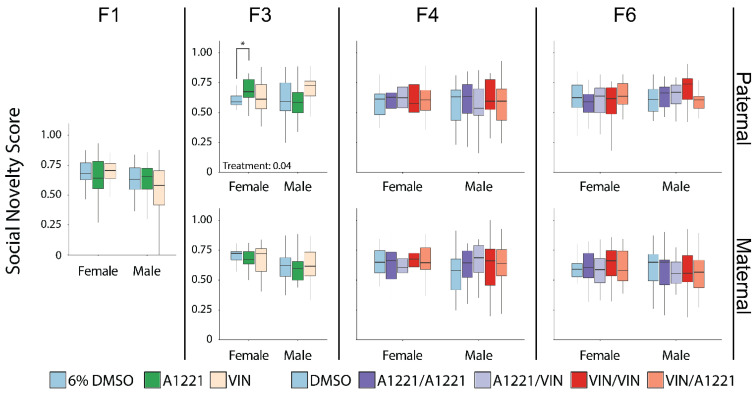
In the social novelty test, the social novelty score (time spent with the novel animal divided by the sum of the time spent with both the novel and familiar stimulus animals) is graphed as box and whisker plots (minimum, 25% quartile, median, 75% quartile, and maximum) shown separately by generation (F1 to F6 from left to right), lineage (paternal—top and maternal—bottom), and sex (indicated on *x*-axis). Lineage does not apply to the F1 generation. The social novelty score was calculated as the time spent near the novel stimulus animal divided by the time spent near the novel animal plus the time spent near the familiar stimulus animal. Scores above 0.50 indicate a preference for socializing with the novel animal. All group means were above the 0.50 threshold. Significant post hoc tests as determined by Tukey’s HSD are indicated with bars connecting the respective groups (* <0.05).

**Figure 7:**
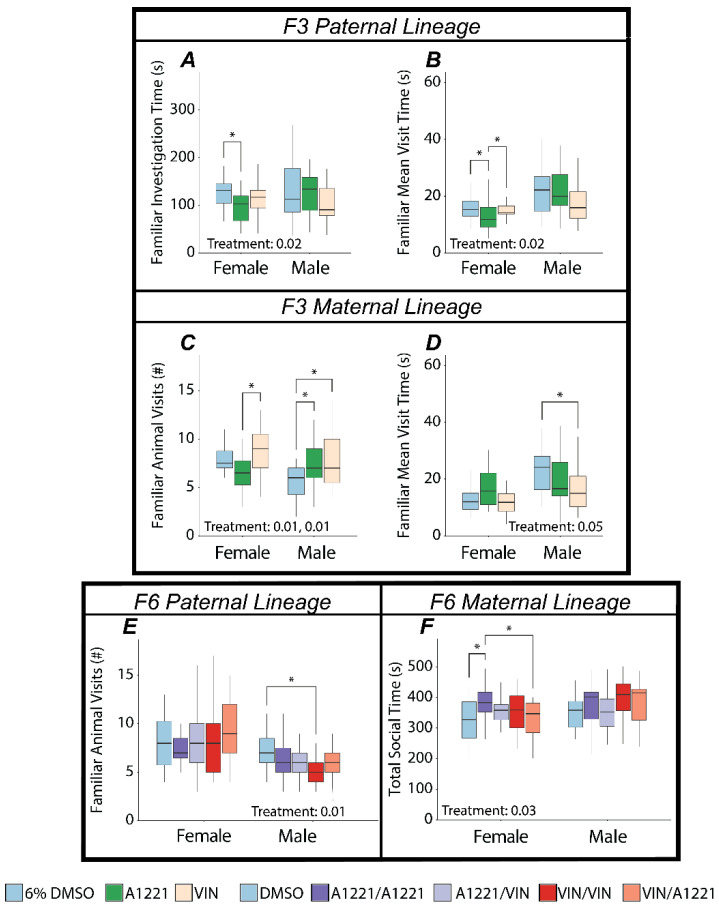
Aspects of social interaction dynamics in the test for social novelty are graphed as box and whisker plots (minimum, 25% quartile, median, 75% quartile, and maximum). Main effects of treatment within sex and lineage were determined by a one-way ANOVA within each sex and lineage, and indicated for *p* < 0.05. Significant post hoc results as determined by Tukey HSD and appropriately adjusted for multiple comparisons are indicated with bars connecting the respective groups (* <0.05). Shown are significant effects for the F3 paternal (**A**,**B**) and maternal (**C**,**D**) lineages, and F6 paternal (**E**) and maternal (**F**) lineages.

In the F1 generation there were no effects of EDC exposure on the social novelty score, defined as the time spent with the novel animal divided by the sum of the time spent with both the novel and familiar stimulus animals. In the F3 generation, the social novelty score was affected by ancestral EDC exposure in females from the paternal lineage (*F*(2,58) = 3.49, *p* = 0.04, η*p*^2^ = 0.11—Figure 6), which was largely driven by an increase in the A1221 group compared to DMSO (*p* = 0.03). This change was associated with an effect of treatment in the amount of time spent with the familiar animal (*F*(2,58) = 4.08, *p* = 0.02, η*p*^2^ = 0.12—Figure 7A), attributable to a decrease in the A1221 group vs. DMSO (*p* = 0.02). Time spent with the novel animal was unaffected. While the number of entries to the familiar stimulus animal chamber was not changed, familiar mean visit time was affected (*F*(2,58) = 4.38, *p* = 0.02, η*p*^2^ = 0.13—Figure 7B), with a decrease in the A1221 group compared to both DMSO (*p* = 0.03) and VIN (*p* = 0.04) ancestral exposure. In the maternal lineage, social novelty score was unaffected, whereas aspects of social interaction were changed in both maternal lineage males and females. Treatment affected the number of visits to the familiar animal in both females (*F*(2,56) = 4.63, *p* = 0.01, η*p*^2^ = 0.14—Figure 7C) and males (*F*(2,66) = 5.60, *p* = 0.01, η*p*^2^ = 0.15—Figure 7C). In females this was driven by a decrease in visits of the A1221 group compared to the VIN group (*p* = 0.01). In males, both A1221 (*p* = 0.05) and VIN (*p* = 0.01) exposed individuals showed an increase in total visits to the stimulus animal compared to DMSO males. Mean visit time of males from the maternal lineage was decreased (*F*(2,66) = 3.19, *p* = 0.05, η*p*^2^ = 0.09—Figure 7D) and driven primarily by a decrease in the VIN group compared to DMSO (*p* = 0.04). The social novelty score was not affected in either sex or breeding lineage in the F4 or F6 generations (Figure 6), nor was there any change in aspects of social interaction.

In F6 paternal lineage males there was an effect of treatment on the number of visits to the familiar animal in males (*F*(4,99) = 3.34, *p* = 0.01, η*p*^2^ = 0.12—Figure 7E). In the F6 maternal lineage, there was an effect on locomotion (distance traveled) in males (*F*(4,98) = 3.21, *p* = 0.02, η*p*^2^ = 0.12—not shown), for which the A1221/VIN treatment group (*p* = 0.01) was increased compared to the VIN/VIN group. This was the only effect of EDC exposure on locomotion in any sex, generation, treatment, or lineage in the social novelty test. In females from the F6 maternal lineage, the only behavioral metric affected was total social time (*F*(4,102) = 2.83, *p* = 0.03, η*p*^2^ = 0.10—Figure 7F) where the A1221/A1221 group spent more time associating with either the familiar or novel animal compared to DMSO (*p* = 0.05) and VIN/A1221 (*p* = 0.03).

### 3.3. Nose-Touching Behaviors

#### 3.3.1. Validation of Machine Learning Accuracy

Three independent validation datasets were used to train, optimize, and verify the automated detection of nose-touch instances with machine learning. The first was used to train the machine learning model to accurately detect and track individual body parts on the experimental animal and the stimulus animals. The accuracy of the model was determined by comparing the distance in pixels between the coordinate location of a manually indicated body part (e.g., a rat’s nose) and the coordinate location of a body part predicted by the model (Figure 3B). Our final model showed a root mean squared error (RMSE) of 6.08 pixels. For comparison, a rat’s nose is approximately 5 pixels long and 5.5 pixels wide. The second validation dataset was used to optimize the parameters that determined what should constitute a nose touch. The same dataset was manually scored for nose touches three separate times by the same blind experimenter and once by the algorithm used to extract nose-touch instances. The human-variability (RMSE) in the first two manually scored sets was 4.98 and 3.54 s when compared to the third. Variability between the third human scored dataset and the final computer model was 4.31 s. Because this second dataset was itself used to optimize the parameters for which nose touches were detected, and therefore could be subject to overfitting, we generated a third manually scored dataset that was also scored by the computer model and compared for accuracy (RMSE = 3.63 s). We found that our method to automate the detection of nose touches was as accurate as a human scorer and generalized well to the entire dataset.

#### 3.3.2. Sex Differences in Nose Touching

Nose touch data were analyzed for sex differences in time spent nose touching, average duration of nose touches, and the longest-duration nose touch (Table 2). A nose touch score was also calculated for social novelty as the amount of time nose touching with the novel animal divided by the sum of the time nose touching with both the familiar and novel stimulus animals. In the Sociability test, males and females spent similar time nose-touching with a stimulus animal (*H*(1) = 1.08, *p* = 0.30, *d* = 0.07) but the average duration of nose touches was longer in females than males (*H*(1) = 9, *p* = 0.003, *d* = 0.07). In social novelty, males and females did not show a difference in nose-touch novelty score (*H*(1) = 0.04, *p* = 0.84, *d* = 0.04) but females spent more total time nose touching with both the familiar (*H*(1) = 8.29, *p* = 0.004, *d* = 0.09) and novel (*H*(1) = 18.71, *p* < 0.0001, *d* = 0.22) stimulus animals.

#### 3.3.3. EDC Effects on Nose Touching

**Figure 8:**
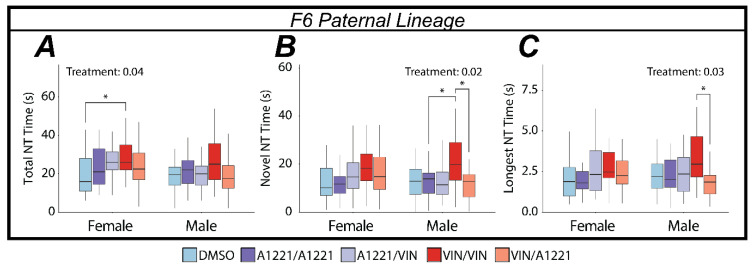
Metrics of nose-touching (NT) in the social novelty test as determined by leveraging machine learning techniques are graphed as box and whisker plots (minimum, 25% quartile, median, 75% quartile, and maximum). Main effects of treatment were determined by a one-way ANOVA within each sex and lineage, and indicated for *p* < 0.05. Significant post hoc results as determined by Tukey’s HSD and appropriately adjusted for multiple comparisons are indicated with bars connecting the respective groups (* <0.05). (**A**) The only nose-touch metric affected in females was identified in the F6 paternal lineage where a combination of an ancestral and direct exposure of VIN increased total nose-touch time (the sum of familiar and stimulus NT time). (**B**,**C**) The time spent nose-touching with the novel stimulus animal and the longest nose-touch interaction were both increased in males from the F6 paternal lineage exposed to an ancestral exposure and a direct exposure of VIN. Taken together, these data suggest ancestral VIN exposure may influence social identification or discrimination.

In the Sociability test, none of the metrics analyzed for nose touches were found to be affected by treatment in either sex or in any of the generations or lineages. In the social novelty test, the effects identified were exclusive to the F6 paternal lineage. In females, the only metric affected by treatment was total nose-touch time (*F*(4,115) = 2.65, *p* = 0.04, η*p*^2^ = 0.08—Figure 8A) in which the VIN/VIN group showed an increase compared to DMSO (*p* = 0.02). In males, the total amount of time spent nose touching with the novel animal was affected by treatment (*F*(4,113) = 3.11, *p* = 0.02, η*p*^2^ = 0.10—Figure 8B); the VIN/VIN group was increased compared to A1221/A1221 (*p* = 0.04) and VIN/A1221 (*p* = 0.02). A more nuanced metric of nose touching (longest-duration nose touch) was also affected (*F*(4,113) = 2.80, *p* = 0.03, η*p*^2^ = 0.10—Figure 8C) with VIN/VIN higher than VIN/A1221 (*p* = 0.01).

## 4. Discussion

Our model of two hits of EDCs given three generations apart enabled us to begin to decipher the combinatorial effects of multigenerational exposures to legacy and contemporary chemicals for the first time. Built into our design, and evident in the results, were concepts that are critical to research on EDCs. First, the sexes respond differently to direct exposures to environmental toxicants, especially during critical developmental periods when hormone release and actions differ between the sexes. Second, epigenetic programming of the germline by EDCs is both sex-specific and dependent upon paternal and maternal lineage. Third, exposures to EDCs within and across generations may have unexpected outcomes.

Another novel aspect of our study was developing and applying machine learning with DeepLabCut to analyze nose-touching behavior in rats. This type of endpoint is important because social recognition happens through species-specific cues that may be obvious to a conspecific but not to a human observer. In rats, this involves close-in facial investigation and the assessment of pheromonal and olfactory cues, alterations of which by EDCs may change the dynamics of social interactions [21,51]. EDCs were observed to change nose-touch behavior and facial investigation between same sex-conspecifics in adulthood [6,8].

Finally, independent of any EDC or lineage effects, our massive behavioral dataset enabled us to thoroughly characterize and directly compare male and female rats with a sample size of ~600 animals per sex. We verified the well-known increased locomotor activity of female over male rats. Previous work showing that males spend more time investigating stimulus animals than females [6,52,53,54] was confirmed here; our males spent more time associating with stimulus animals in both the sociability and social novelty tasks. While males spent more time with a stimulus animal, females made more visits to each stimulus animal but for shorter duration bouts than males. Despite spending less time with stimulus rats, females had a stronger preference for social novelty than males. These comparisons provide a strong baseline for other studies on sex differences in social behaviors.

### 4.1. Direct Developmental Exposure to EDCs (F1 Generation) Have Few Effects on Social Behaviors

The effects of direct EDC exposure on social behavior have been reported in several studies, with outcomes dependent on the compound used, the timing of exposure, the age at testing, and the endpoints measured. To date, effects of direct EDC exposure on social behaviors have been reported for PCBs [5,6], BPA [7,8], atrazine [7], phthalates [9,10], chlorpyrifos [11], and vinclozolin [12,55]. These experiments show that EDC exposures alter subsets of behavior, and that expected sexual dimorphisms of behavior are sometimes diminished [14].

Our current study did not find any effects of direct A1221 or VIN exposure on social behaviors in the F1 generation. These results were surprising based on this prior literature, but the timing and doses of treatment used here differed from previous work. EDCs often exert nonmonotonic dose-response curves [56], and these effects are further influenced by the developmental stage. The fact that we did not observe direct EDC effects on social behaviors, but that effects were found in subsequent generations, suggests that the selected doses and timing may not be adequate to causes direct developmental changes but were still able to induce heritable epigenetic profiles through actions on the germline, as demonstrated previously [57] with the same dose and exposure paradigm used here.

### 4.2. EDCs Affect Social Behavior in Ancestrally Exposed Individuals

Studies on effects of ancestral exposure to EDCs on social behavior have demonstrated perturbations in the F3 generation. Transgenerational BPA increased social behavior and impaired dishabituation of social novelty in females [15,16]. Transgenerational exposure to antiandrogenic EDCs such as phthalates [17,18] and VIN [19] reduced social behaviors in males. In all of these studies, different doses and behavioral paradigms makes comparing the results between them and discerning the differential impact of EDC classes difficult. However, it is particularly noteworthy that the doses used here (1 mg/kg/day A1221 or VIN) are much lower than our previous work (e.g., 100 mg/kg [19,31]), better represent real-world exposures, and still produce robust heritable transgenerational phenotypes. Here, we directly compared two different classes of EDCs, and their combination, across generations to determine how social behavior was affected.

#### 4.2.1. The Paternal Lineage Females Are Most Vulnerable to Ancestral Exposure

It has been proposed the male germline is particularly susceptible to environmental input and insult [58] and there are numerous demonstrations that the male germline is directly affected by EDC exposure (reviewed in [4]). Evidence for the female germline is more limited, probably due to the much greater ease in isolating and purifying sperm compared to ova. This has led to bias in work considering transgenerational endpoints in paternal descendants compared to studies on the maternal lineage. Our current study is the most comprehensive in its consideration of lineage of origin and sex due to EDC exposure. We found that ancestral EDC exposure effects on social behavior were more frequent in the paternal lineage, but there were also some maternal lineage effects.

Among the behaviors we analyzed, rats’ preference for a conspecific over an empty cage (Sociability), and the preference for a novel over a familiar rat (social novelty), are most relevant to social decision-making in rodents and are discussed here. We found a main effect of treatment in three comparisons, all of which were in females from the paternal lineage: ancestral VIN exposure decreased sociability in the F3 and F6 generations while ancestral A1221 exposure increased the preference for social novelty in the F3 generation. There are three primary points to take away from these results. First, female social behavior is particularly susceptible to EDC exposure, with EDC class (estrogenic vs. antiandrogenic) playing a role. Second, altered social behavior emerges in the F3 generation, presumably due to direct exposure of the F2 germline. Finally, the paternal germline is more susceptible than the maternal to EDC exposure in the context of social behavior, although we emphasize that this result should not be extrapolated to other endpoints affected by EDCs.

It is notable that those rats of the F4 generation receiving a second hit of the same EDC (i.e., A1221/A1221 or VIN/VIN) differed in their behaviors from those receiving a single hit. There are several possible interpretations, including that germline perturbations are corrected or diminished, or that there is an interaction between ancestral exposure and direct exposure whereby one mitigates the other. This remains to be determined. There were, however, aspects of social interaction dynamics altered in F4 rats, in which A1221 decreased the number of visits to an animal but increased the duration of those visits in the paternal lineage.

#### 4.2.2. PCBs Are an Underappreciated Agent of Transgenerational Perturbations

A recent review on the transgenerational impact of EDC exposure on transgenerational endpoints included 43 primary research articles, of which 17 were focused on VIN and only one (from our research group) reported results on PCBs [4]. Our lab subsequently published a second article on transgenerational A1221 effects [59]. PCBs are well described for their endocrine disrupting actions when exposure occurs during development [1] but the literature on the transgenerational effects of PCBs on behavioral or molecular endpoints is limited. We previously showed that A1221 induced heritable epimutations in both F3 sperm and brain [57]; increased body weight, circulating progesterone, and estradiol [60], and caused aberrant gene expression in the hypothalamus in the F3 generation [59]. The current study adds to the transgenerational literature with results showing that A1221 increased preference for social novelty in paternal F3 females, an effect that was not present in the F1 generation, nor did it persist to the F4 or F6 generations after an additional hit of A1221. Two hits of A1221 also did not change the overall preference for social novelty but induced changes in social interaction dynamics (nonsocial time and average visit time) in the F4 generation. These effects again did not persist to the F6 generation; they also did not occur in groups where the first hit or the second hit were different EDCs. These effects demonstrate that A1221 is an agent of heritable behavioral change, at least in the context of social behavior.

#### 4.2.3. The Order of EDC Exposure Is Important

A1221 represents an EDC class (PCBs) that is no longer manufactured, but persists in our environment. VIN represents an EDC that was introduced about five decades after A1221 and is still in agricultural use. Based on evidence that A1221 and VIN alter unique subsets of differentially methylated regions in F3 sperm [57] we hypothesized that associated behavioral phenotypes would differ. The present data confirm that hypothesis. A number of effects were dependent on the order of the EDC exposure; for example, A1221/VIN females from the paternal F6 generation had increased preference for social affiliation when compared to VIN/VIN females. While neither of these groups were determined to be different from the control group, these data show that the changes caused by specific EDCs can set a trajectory that can either amplify or diminish further exposures.

We are not aware of any experimental precedence to these findings, so putting these results into context is difficult. However, we strongly believe that future experiments considering heritable transgenerational phenotypes should try to include historically relevant exposure models that might also include complex EDC mixtures so that we can more realistically model human and wildlife exposures.

## Figures and Tables

**Table 1:** Sample sizes, indicated as # litters/# individual females/# individual males.

	**F1**	**F3**					
First Hit		***Maternal***	***Paternal***				
**DMSO**	10/21/25	11/21/21	10/23/21				
**A1221**	10/28/24	10/18/24	11/22/18				
**VIN**	10/28/24	12/19/23	11/18/21				
				**F4**		**F6**	
First Hit/Second Hit				***Maternal***	***Paternal***	***Maternal***	***Paternal***
**DMSO/DMSO**				13/22/21	12/25/22	12/22/23	12/21/24
**A1221/A1221**				9/15/18	8/18/15	9/18/20	8/18/20
**A1221/VIN**				9/18/19	9/17/18	9/22/18	9/20/20
**VIN/A1221**				12/21/22	11/21/21	11/19/23	11/22/21
**VIN/VIN**				13/20/22	12/19/20	12/22/22	10/19/22

Note: The total number of litters, individual females, and individual males used in this study are shown, with treatment, generation, and lineage indicated. On average, 10 litters were used per group and in most cases 2 male and 2 female individuals from each litter were behaviorally characterized, although those groups with fewer litters (due to timeline limitations) occasionally included a third individual per sex.

**Table 2:** Sex differences in the sociability and social novelty tests.

**Sociability**				
	**Females**		**Males**	
**Measure**	**Mean**	**SEM**	**Mean**	**SEM**
Distance Traveled (m) *	52.63	0.36	38.58	0.3
Social Preference Score *	0.62	0.01	0.67	0.01
Stimulus Investigation Time (s) *	224.35	2.68	245.90	3.02
Empty Investigation Time (s) *	135.26	2.13	123.31	2.59
Stimulus Animal # Visits *	10.26	0.13	8.68	0.14
Empty Chamber # Visits *	8.42	0.11	6.25	0.09
Stimulus Animal Mean Visit Time (s) *	24.46	0.5	34.76	1.06
Empty Chamber Mean Visit Time (s) *	17.58	0.38	22.78	0.68
Total Nonsocial Time (s) *	240.01	2.79	229.86	2.86
Center Isolation Time (s) *	99.54	1.19	107.72	1.6
Total Nose-Touch Time (s)	20.21	0.40	19.51	0.39
Average Nose-Touch Duration (s) *	0.66	0.01	0.63	0.01
**Social Novelty**				
	**Females**		**Males**	
**Measure**	**Mean**	**SEM**	**Mean**	**SEM**
Distance Traveled (m) *	46.35	0.39	32.44	0.29
Social Novelty Score *	0.62	0.01	0.60	0.01
Familiar Investigation Time (s) *	124.31	2.16	142.47	2.84
Novel Investigation Time (s)	208.61	2.81	215.03	3.17
Familiar Animal # Visits *	8.01	0.11	6.32	0.09
Novel Animal # Visits *	10.09	0.13	7.74	0.11
Familiar Animal Mean Visit Time (s) *	16.89	0.55	24.67	0.6
Novel Animal Mean Visit Time (s) *	22.46	0.44	31.39	0.72
Total Social Time (s) *	332.92	2.96	357.50	3.16
Total Nonsocial Time (s) *	273.37	3.77	249.89	4.04
Center Isolation Time (s) *	125.54	1.78	116.37	1.92
Nose-Touch Novelty Score	0.65	0.01	0.64	0.01
Familiar Animal Nose-Touch Time (s) *	9.33	0.27	8.62	0.30
Novel Animal Nose-Touch Time (s) *	17.77	0.41	15.41	0.38

Note: Sex differences in sociability (top) and social novelty (bottom) are shown for all animals with treatment, generation, and lineage collapsed. Mean and standard error of the mean (SEM) are shown for females (N = 596) and males (N = 601). These summary data provide a definitive comparison of sex differences for the most important measures from the sociability (Top) and social novelty (Bottom) behavioral tasks. Nearly all metrics observed were sexually dimorphic and are indicated by * where *p* < 0.001. Of greatest relevance to the individual tasks, males displayed a stronger social preference score (Top) but females showed a stronger social novelty score (Bottom). #: number of events.

## Data Availability

Data will be made available upon request.
